# Comparing microbial community compositions of biogas and sewage treatment plants by analyzing 16S rRNA gene data

**DOI:** 10.1016/j.dib.2018.09.118

**Published:** 2018-10-04

**Authors:** Christian Buettner, Matthias Noll

**Affiliations:** Coburg University of Applied Sciences and Arts, Institute for Bioanalysis, Friedrich-Streib-Straße 2, D-96450 Coburg, Germany

## Abstract

This article contains data of bacterial and archaeal community compositions and process parameters of 16 biogas (BPs) and ten sewage treatment plants (STPs). Nucleic acids of BPs were extracted using a CTAB-based method while a phenol-chloroform-based method was applied for STPs. Amplicon sequencing data of the 16S rRNA gene was achieved by MiSeq-technology. Raw data were processed and statistically analyzed by several approaches including network analyses by using molecular ecological network analysis (MENA). Nodes of each network (BPs and STPs) were classified as generalists and peripherals to identify key players for a stable biogas production. Network parameters as well as interaction of generalistic species were compared between both plant types.

## Specifications table

**Table:** 

Subject area	*Environmental Sciences*
More specific subject area	*Molecular Ecology, Interaction of microbial communities*
Type of data	*Two figures, nine tables and one entry into SRA (merged reads)*
How data was acquired	*Nucleic acid extraction of samples was followed by 16S rRNA gene analysis using MiSeq-technology. Raw data were processed and network analysis using Molecular Ecological network analysis (MENA) was applied.*
Data format	*Raw, processed, filtered, analyzed*
Experimental factors	*16 biogas plants and ten sewage treatment plants were sampled in August 2016. Important process parameters of each plant were recorded.*
Experimental features	*DNA was extracted using plant-specific methods. Sequencing of DNA extracts was done using MiSeq-technology. Data were processed and analyzed using network analysis and other methods.*
Data source location	*All plants are located in Northern Bavaria (Germany).*
Data accessibility	*All tables and figures are within this article and supplementary material except for merged sequence reads. They are stored in the SRA of NCBI (ID:*SRP126305) *and are accessible under following URL:*https://www.ncbi.nlm.nih.gov/sra/SRP126305

## Value of the data

• Surveys of bacterial and archaeal community compositions of many full-scale anaerobic digestion systems are still lacking. This issue is tackled in this article.
• Process parameters of at least ten plants of each plant type were documented, enabling a solid comparison of process parameters and bacterial and archaeal community compositions.
• Primers (515YF and 806R) are common, which ease data comparisons of this article with other data sets using similar methods.
• Data include potential new bacterial as well as archaeal interaction patterns that could be further investigated in co-culturing experiments or other experimental set-ups.

## 1 Data

Paired-end sequence reads were produced by PCR-generated amplicons of the V4 variable regions of the 16S rRNA gene on an Illumina MiSeq. Reads were processed and finally merged by a minimum overlap of 15 base pairs and OTUs were clustered using an identity threshold of 99%.

Filtered, absolute abundances of OTUs were thereafter used for calculating a variety of diversity indices, a Bray–Curtis similarity heatmap and network analyses.

## 2 Experimental design, materials and methods

### 2.1 Sampling and DNA-extraction

16 biogas plants (BPs) and digestions tanks of 10 sewage treatment plants (STPs) were sampled in August 2016. Prior to sampling, pipelines were flushed and sludge was discarded. Afterwards, sludge was mixed thoroughly and samples were shock-frozen in liquid nitrogen and stored at − 80 °C. Available plant parameters were collected from the plant operators (Tables 1 and 2). Different methods for DNA-extraction were tested. Nucleic acids from sludge of BPs and of STPs was finally extracted according to a modified CTAB-based method [1] and a modified phenol-chloroform-based method [2], respectively. Detailed description of each method can be found elsewhere [3].

**Table 1:** Recorded process parameters of sampled STPs.

**Plant**	**Capacity [PE]**	**Waste types**^b^	**pH**	**Temp. [°C]**	**COD [mg/L]**	**BOD**_**5**_**[mg/L]**	**SRT [d]**	**Substrate treatment**	**STY**^a^	**Mixing**
STP01	30,000	1	7.00	35	20	3	33–35	mechanical	0.16	continuous
STP02	40,000	1	7.00	36	350	100	45	mechanical	0.25	continuous
STP03	62,500	1,2	7.00	37	45	20	60	enzymatic	0.20	discontinuous
STP04	300,000	1, 4, 5	7.05	35	18	2	28	enzymatic	0.27	continuous
STP05	25,000	1	7.10	37	467	224	100	mechanical	0.26	continuous
STP06	300,000	1, 3 ,4, 5	7.20	41	752	437	33	enzymatic	0.64	continuous
STP07	220,000	1, 3 ,4, 5	7.25	40	890	440	18–21	ultrasonic	0.80	continuous
STP08	90,000	1	7.30	37	654	394	44	mechanical and enzymatic	0.28	continuous
STP09	35,000	1	7.50	37	365	212	55–60	mechanical	0.27	continuous
STP10	290,000	1, 2, 3, 4	7.65	39	618	305	21	none	1.00	continuous

a Space-Time-Yield biogas plant: m^3^/day; fermenter: volume/day.

b 1: municipal waste; 2: textile industry; 3: food industry; 4: brewing industry; 5: slaughterhouse waste.

**Table 2:** Recorded process parameters of sampled BPs.

**Plant**	**Temp. [°C]**	**pH**	**Substrate proportions [%]**^a^^**,**^^b^								**Fermentation type**	**Mixing**	**Substrate pretreatment**
			**1**	**2**	**3**	**4**	**5**	**6**	**7**	**8**			
**BP01**	38	7.5	83	1	6	10	0	0	0	0	wet	discontinuous	thermal
**BP02**	38	7.6	78	0	0	22	0	0	0	0	wet	discontinuous	none
**BP03**	42	7.7	85	0	0	0	0	0	15	0	wet	discontinuous	none
**BP04**	42	7.7	32	44	16	2	0	0	2	4	wet	discontinuous	none
**BP05**	42	7.9	0	0	35	0	35	28	0	2	dry	continuous	none
**BP06**	43	8.0	0	0	0	0	80	0	0	20	wet	continuous	mechanical
**BP07**	43	7.9	25	0	0	0	40	15	10	10	wet	discontinuous	mechanical
**BP08**	43	7.7	25	0	0	0	35	20	0	25	wet	discontinuous	mechanical
**BP09**	45	7.8	65	0	0	0	25	10	0	0	wet	continuous	none
**BP10**	45	8.1	20	15	0	0	20	0	40	5	wet	continuous	none
**BP11**	45	7.8	35	0	0	0	55	5	0	5	wet	discontinuous	none
**BP12**	45	7.7	40	0	5	0	40	10	5	0	wet	continuous	none
**BP13**	45	7.7	30	0	0	0	30	15	10	15	wet	discontinuous	none
**BP14**	45	7.7	45	0	0	0	35	10	0	10	wet	continuous	enzymatic
**BP15**	54	8.0	48	0	3	0	32	2	13	2	wet	discontinuous	none
**BP16**	60	7.5	0	0	0	100	0	0	0	0	wet	continuous	thermal

a 1: cattle slurry; 2: pig slurry; 3: solid manure; 4: food residues; 5: maize silage; 6: grass silage; 7: whole crop silage; 8: crop silage.

b Average values are given, because sampled plants usually operating with a stable substrate supply.

### 2.2 Generating and processing the raw data of 16S rRNA gene sequences

Nucleic acid extracts obtained from each STP and BP plant were amplified by a two-Step PCR using the primer set 515F and 806R (details see [3]) and sequence libraries were created by v2 500 cycles kit (Illumina, San Diego, CA, USA) according to manufacturer׳s instructions. Sequencing of the libraries was carried out by 300-bp paired-end sequencing on an Illumina MiSeq system. Raw data were de-multiplexed, quality filtered and adaptor trimmed using the Illumina real time analysis software (Illumina, San Diego, CA, USA). Quality of the reads was checked with the FastQC software, version 0.11.5 [4]. Adaptors were trimmed using the software cutadapt v.1.9.2.dev0 [5]. Reads that could not be trimmed were discarded. Merging of the trimmed forward and reverse reads, considering a minimum overlap of 15 bases, was done by using USEARCH version 8.1.1861 [6]. Clustering of OTUs with a 99% similarity level, discarding singletons and chimeras, was performed using USEARCH. Taxonomic assignment of OTUs was done with an identity threshold of 0.7 against the Greengenes v13.8 database [7]. Finally, an OTU table of absolute abundances of each OTU for each plant was generated. All steps mentioned above were performed by Microsynth AG (Balgach, Switzerland). Processed, merged reads were stored in the Short Read Archive of the NCBI (ID: SRP126305) and are accessible under following URL: https://www.ncbi.nlm.nih.gov/sra/SRP126305.

### 2.3 Data analyses of processed 16S rRNA gene data sets

**Fig. 1:**
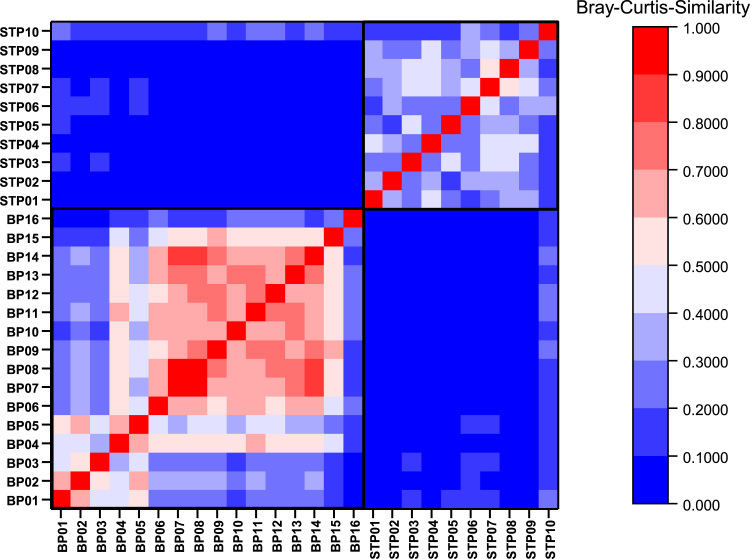
Heatmap of the Bray–Curtis similarity between all plants. For better comparability between both plant types (BPs and STPs), similarity measures are based on the unfiltered absolute abundance of each OTU. Dark blue indicates a low similarity (= 0), while strong red indicate a high similarity (= 1). Similarity matrices of BPs and STPs are separated by black boxes.

Total number of sequence reads, number of bacterial and archaeal sequence reads as well as archaeal proportions of reads were summarized (Table 3). Generated OTU table was used as input matrix to generate a Bray–Curtis similarity matrix using function vegdist of R-package vegan v.2.4-3 [8]. Resulting matrix was visualized using Origin 2017 (OriginLab Corporation, Northampton, MA, USA) enabling a comparison of both bacterial and archaeal community composition between biogas and sewage treatment plants (Fig. 1).

**Table 3:** Amount of sequence reads assigned to the kingdom *Archaea* and *Bacteria*. Number of corresponding OTUs with a relative abundance > 0.1% is given in brackets.

**Plant**	**Reads of*****Archaea***	**Reads of*****Bacteria***	**Total number of reads**	**Relative proportion of*****Archaea*****[%]**
**BP01**	12,582 (25)	502,361 (339)	514,943 (364)	2.44
**BP02**	32,702 (27)	505,690 (340)	538,392 (367)	6.07
**BP03**	10,008 (25)	265,081 (323)	275,089 (348)	3.64
**BP04**	3428 (18)	601,985 (316)	605,413 (334)	0.57
**BP05**	5174 (23)	568,265 (344)	573,439 (367)	0.90
**BP06**	444 (23)	660,621 (271)	661,065 (294)	0.07
**BP07**	1453 (23)	533,752 (322)	535,205 (345)	0.27
**BP08**	1165 (32)	479,242 (292)	480,407 (324)	0.24
**BP09**	4227 (28)	351,973 (286)	356,200 (314)	1.19
**BP10**	823 (32)	554,132 (305)	554,955 (337)	0.15
**BP11**	2842 (19)	447,107 (317)	449,949 (336)	0.63
**BP12**	3580 (19)	361,370 (298)	364,950 (317)	0.98
**BP13**	3915 (21)	566,967 (291)	570,882 (312)	0.69
**BP14**	2763 (27)	550,834 (291)	553,597 (318)	0.50
**BP15**	1763 (22)	339,115 (266)	340,878 (288)	0.52
**BP16**	677 (27)	334,310 (277)	334,987 (304)	0.20
**BPs total**	**Σ = 87,546**	**Σ = 7,622,805**	**Σ = 7,710,351**	***Ø* = 1,19 ± 1.60**
**STP01**	17,551 (29)	340,647 (478)	358,198 (507)	4.90
**STP02**	14,264 (29)	253,457 (435)	267,721 (464)	5.33
**STP03**	34,710 (32)	396,040 (452)	430,750 (484)	8.06
**STP04**	29,478 (32)	355,093 (447)	384,571 (479)	7.67
**STP05**	15,841 (24)	337,406 (431)	353,247 (455)	4.48
**STP06**	15,259 (28)	449,841 (412)	465,100 (440)	3.28
**STP07**	9090 (29)	327,042 (428)	336,132 (457)	2.70
**STP08**	13,630 (31)	238,889 (441)	252,519 (472)	5.40
**STP09**	14,681 (28)	351,588 (416)	366,269 (444)	4.01
**STP10**	9582 (30)	374,492 (422)	384,074 (452)	2.49
**STPs total**	**Σ = 174,086**	**Σ = 3,424,495**	**Σ = 3,598,581**	***Ø* = 4.83 ± 1.89**

Based on the results of Bray–Curtis similarity heatmap, both plant types (BPs and STPs) were thereafter separately analyzed. Relative abundances of each OTU were calculated for each plant. OTUs below a minimum relative abundance of 0.1% in at least one plant of respective plant type were discarded. To avoid loss of too many archaeal OTUs, total relative abundance of bacterial and archaeal sequence reads were separated and each data set was set to 100%. Relative abundances of remaining OTUs were re-normalized to 100% and filtered OTU tables for both groups were generated (Supplementary Tables 5 and 6). Absolute abundances of remaining OTUs were then used for calculating alpha diversity measurements (OTU richness, Shannon, Simpson (1-D)) and OTU richness estimators (Chao1, abundance based coverage estimator (ACE)) using R-package vegan v.2.4-3 [8] (Table 4). Significance between mean values of both plant types was calculated using Mann–Whitney U test. Additionally, filtered OTU tables were summarized up to family level (Supplementary Tables 7 and 8) for a less complex overview of community compositions.

**Table 4:** Diversity indices for BPs and STPs of OTUs with a relative abundance ≥ 0.1%. The significance thresholds were defined as *p* ≤ 0.001 (**) and *p* ≤ 0.0001 (***).

**Plant**	OTU richness***	Shannon***	Simpson (1-D)**	Chao1***	ACE***
**BP01**	364	4.456	0.974	371.333	370.720
**BP02**	367	4.529	0.978	373.000	375.972
**BP03**	348	4.635	0.978	351.500	351.002
**BP04**	334	3.901	0.941	359.667	350.877
**BP05**	367	4.213	0.961	373.875	374.538
**BP06**	294	3.438	0.918	312.474	313.502
**BP07**	345	3.256	0.888	376.500	360.345
**BP08**	324	3.248	0.887	352.050	351.118
**BP09**	314	3.320	0.895	353.048	352.804
**BP10**	337	3.392	0.918	365.636	360.112
**BP11**	336	3.583	0.919	346.222	352.364
**BP12**	317	3.548	0.929	344.444	349.786
**BP13**	312	3.255	0.916	331.500	331.192
**BP14**	318	3.092	0.869	347.176	346.096
**BP15**	288	2.812	0.844	305.647	302.537
**BP16**	304	2.364	0.760	344.182	344.976
**BP mean**	**329.3 ± 24.8**	**3.565 ± 0.634**	**0.910 ± 0.056**	**350.516 ± 20.673**	**349.246 ± 19.836**
**STP01**	507	4.780	0.985	530.917	536.289
**STP02**	464	4.560	0.976	497.300	489.266
**STP03**	484	4.395	0.973	515.714	513.034
**STP04**	479	4.811	0.986	486.286	489.097
**STP05**	455	4.526	0.972	500.000	483.924
**STP06**	440	4.340	0.969	506.000	482.143
**STP07**	457	4.572	0.980	464.286	466.925
**STP08**	472	4.611	0.979	493.667	486.004
**STP09**	444	4.250	0.952	481.000	475.047
**STP10**	452	4.058	0.947	517.045	493.091
**STP mean**	**465.4 ± 20.5**	**4.490 ± 0.233**	**0.972 ± 0.013**	**499.221 ± 19.423**	**491.482 ± 19.812**

### 2.4 Network analyses

**Fig. 2:**
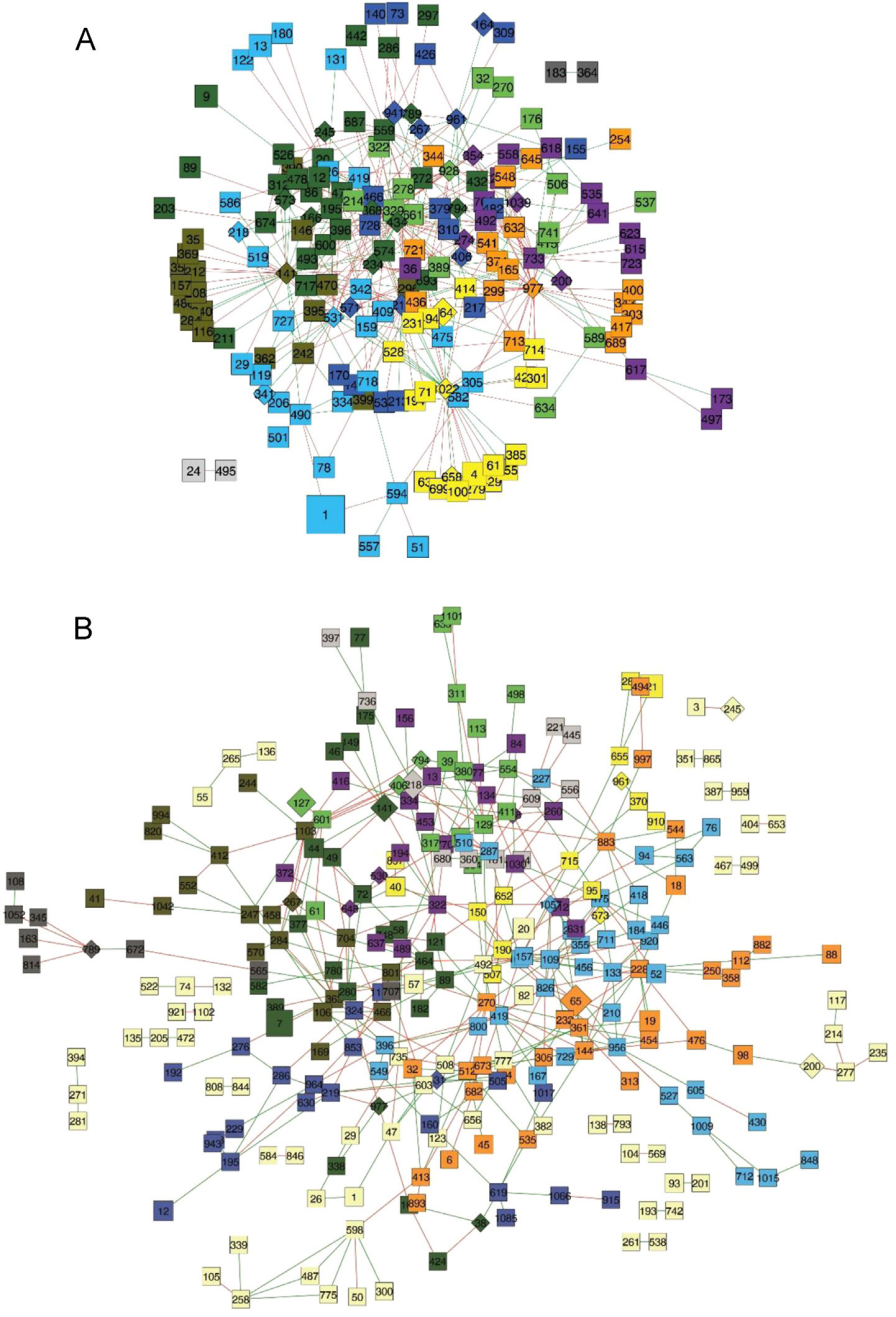
OTU network analysis of sampled plant communities in BPs (A) and STPs (B). Each node represents an OTU and the inlying number indicate the phylogenetic assignment. Archaeal OTUs are denoted as diamonds, bacterial OTUs as squares. Size of the node is dependent of the sum of relative abundances in respective plant type. Node color displays the membership of an OTU to a module. In STP network all modules with less than 10 members are colored in light yellow. Positive interactions are indicated by green connections, while negative interactions are indicated by red connections. List of OTUs to the respective numbers can be found in Supplementary Tables 4 and 5.

Network analysis was carried out separately for each plant group. Filtered relative abundances were used as an input for Molecular Ecological Network Analysis Pipeline [9] (accessible under http://ieg4.rccc.ou.edu/mena/). Network was created based on Spearman similarity matrices. For comparable similarity thresholds of the Spearman similarity matrices, majority for BPs was set to four, while majority of STPs was chosen to be five. Modules were calculated by using fast greedy modularity optimization as described elsewhere [9]. Calculated networks (Fig. 2) were visualized with Cytoscape-software version 3.4.0 [10]. According to Maslov–Sneppen procedure [11], 100 random networks for each of the both networks were calculated for statistical assessment. Topological roles of each node were assigned calculating *P*_i_- (among-module connectivity) and *z*-values (within-module connectivity) following the simplified version [12] of the original definition [13]. Therefore, Peripheral nodes (specialists) were defined by *z* ≤ 2.5 and *P*_i_ ≤ 0.62, connectors by *z* ≤ 2.5 and *P*_i_ > 0.62, module hubs by *z* > 2.5 and *P*_i_ ≤ 0.62 and network hubs by *z* > 2.5 and *P*_i_ > 0.62 (columns G–I of Supplementary Tables 5 and 6). Based on their putative metabolic abilities each generalistic OTU was assigned to one or more steps of anaerobic degradation. Considering that generalists are more important for network functionality than specialists [9], the focus was laid on interactions between generalistic OTUs. To avoid misinterpretations, role during anaerobic degradation of OTUs with insufficient taxonomic assignment was classified as unknown. Finally, a matrix was generated indicating the number of OTU interactions in function of their contribution in anaerobic degradation (Supplementary Table 9).
