# Supplementary material for: Comparing microbial community compositions of biogas and sewage treatment plants by analyzing 16S rRNA gene data
Source: Data Brief. 2018 Oct 4;21:395–402. doi: 10.1016/j.dib.2018.09.118 (PMC6197571; doi:10.1016/j.dib.2018.09.118)
Supplement: Supplementary file 1 — Supplementary material [file mmc1.docx]

Prof. Dr. Matthias Noll July 4th, 2018

University of Applied Science and Arts

Bioanalysis

Friedrich-Streib-Str. 2, 96450 Coburg, Germany

Tel.: ++49 9561 317645; Fax.: ++49 9561 317346

E-Mail: Matthias.noll@hs-coburg.de

**Declaration of interest**

Dear Managing Editor,

All authors declare that there are no competing interests. In addition, the authors certify that we have no affiliations with or involvement in any organization or entity with any financial interest or non-financial interest in the subject matter or materials discussed in this manuscript.

Yours sincerely,

Matthias Noll
